# Comparative study reveals better far-red fluorescent protein for whole body imaging

**DOI:** 10.1038/srep10332

**Published:** 2015-06-02

**Authors:** K.E. Luker, P. Pata, I.I. Shemiakina, A. Pereverzeva, A.C. Stacer, D.S. Shcherbo, V.Z. Pletnev, M. Skolnaja, K.A. Lukyanov, G.D. Luker, I. Pata, D.M. Chudakov

**Affiliations:** 1 Department of Radiology, University of Michigan Medical School, Ann Arbor, MI48109-2200, USA; 2 Tallinn University of Technology, Department of Gene Technology. 15 Akadeemia St, Tallinn 12618, Estonia; 3 Shemiakin-Ovchinnikov Institute of Bioorganic Chemistry, Russian Academy of Science, Miklukho-Maklaya 16/10, 117997, Moscow, Russia; 4 Evrogen JSC, Miklukho-Maklaya 16/10, 117997, Moscow, Russia; 5 Nizhny Novgorod State Medical Academy, Nizhny Novgorod, Russia; 6 CEITEC MU, Masaryk University, Brno, Czech republic; 7 Pirogov Russian National Research Medical University, 117997 Moscow, Russia

## Abstract

Genetically encoded far-red and near-infrared fluorescent proteins enable efficient imaging in studies of tumorigenesis, embryogenesis, and inflammation in model animals. Here we report comparative testing of available GFP-like far-red fluorescent proteins along with a modified protein, named Katushka2S, and near-infrared bacterial phytochrome-based markers. We compare fluorescence signal and signal-to-noise ratio at various excitation wavelength and emission filter combinations using transiently transfected cell implants in mice, providing a basis for rational choice of optimal marker(s) for *in vivo* imaging studies. We demonstrate that the signals of various far-red fluorescent proteins can be spectrally unmixed based on different signal-to-noise ratios in different channels, providing the straightforward possibility of multiplexed imaging with standard equipment. Katushka2S produced the brightest and fastest maturing fluorescence in all experimental setups. At the same time, signal-to-noise ratios for Katushka2S and near-infrared bacterial phytochrome, iRFP720 were comparable in their optimal channels. Distinct spectral and genetic characteristics suggest this pair of a far-red and a near-infrared fluorescent protein as an optimal combination for dual color, whole body imaging studies in model animals.

Whole body fluorescence imaging studies *in vivo* have been enhanced dramatically by development of GFP-like far-red fluorescent proteins^1^,^2^,^3^,^4^,^5^,^6^. Even more options now exist as investigators have introduced a new generation of bacterial phytochrome-based fluorescent proteins that emit near-infrared light^7^,^8^. However, there has been a lack of accurate comparative analysis that would help investigators to select optimal fluorescent proteins as reporters for their current animal imaging studies.

Here we used transfected cell implants in mice to compare previously published GFP-like far-red fluorescent proteins and a modified version of Katushka^1^, named Katushka2S. The latter protein, representing a non-tandem version of td-Katushka2^9^ with elimination of a cryptic donor splice site^10^, demonstrated advanced brightness in preliminary comparative tests in bacterial and eukaryotic cells (data not shown).

## Results

### Comparison *in vitro*

First, we compared characteristics of E2-Crimson, mNeptune, eqFP650, eqFP670, Katushka, and Katushka2S. Key characteristics of the six purified proteins were measured in standard *in vitro* assays (**Methods**). Among tested proteins, Katushka2S exhibited highest brightness and fastest maturation (Table 1). Compared to Katushka, Katushka2S demonstrated higher fluorescence quantum yield and molar extinction coefficient, cumulatively resulting in 33% higher calculated brightness. It should be noted that Katushka2S demonstrated lower photostability than Katushka. However, this feature plays a less significant role in whole body imaging, where scattering and absorption of light naturally limit excitation power^11^.

### Comparison in cultured cells

Comparison of transiently transfected HEK293FT cells with fluorescent signal normalized for transfection efficiency by the IRES-driven luciferase activity on the imaging system IVIS Lumina II (PerkinElmer) also showed superior signal-to-noise ratio from Katushka2S in both “DsRed” (575–650 nm) and “Cy5.5” (695–770 nm) emission channels using excitation wavelengths from 500 to 600 nm (Table 2, Supplementary Fig. 1, Supplementary Table 1). All tested proteins demonstrated low signal-to-noise ratio at 640 nm excitation wavelength making it unfavorable for whole body imaging with GFP-like far-red fluorescent proteins.

### Comparison in whole body imaging

**Figure 1:**
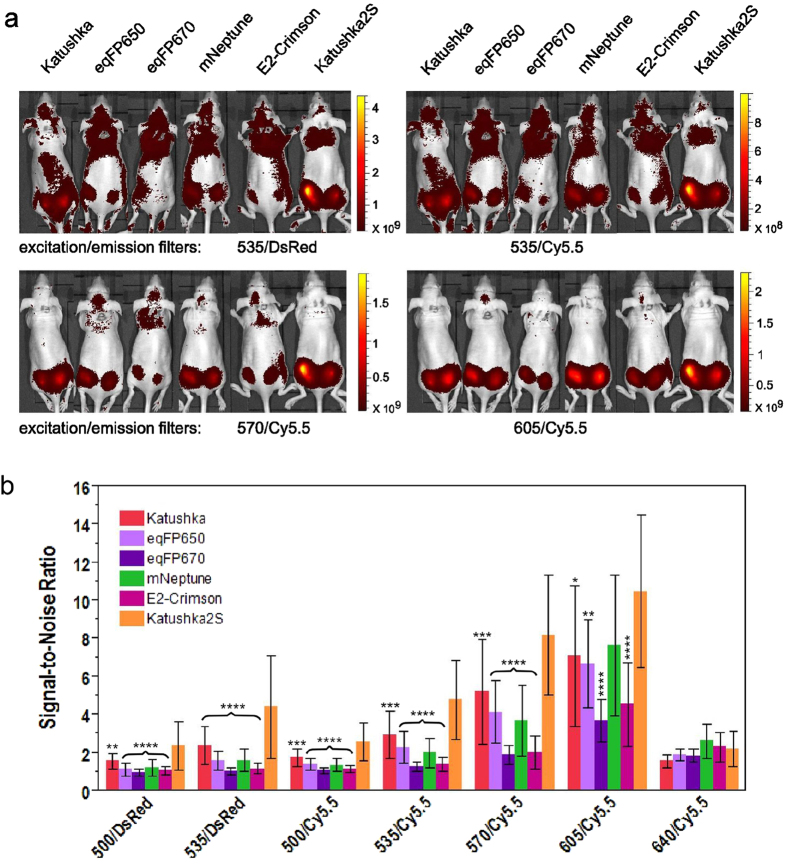
Comparison of far-red fluorescent proteins in whole-mouse imaging. (**a**) Representative fluorescence images of nude mice injected into the gluteal muscle with HEK293FT cells transiently expressing Katushka, eqFP650, eqFP670, mNeptune, E2-Crimson or Katushka2S together with IRES-driven luciferase, captured with indicated excitation and emission filter combinations on IVIS Lumina II. Prior to injection, cells were normalized for transfection efficiency with luciferase activity. Excitation filters are of 35-nm bandwidth centered at the wavelength indicated, and emission filters DsRed and Cy5.5 are 575–650 and 695–770 nm, respectively. Pseudocolor scale bar: radiant efficiency (photons/s)/(μW/cm^2^). Note that scale bars differ for images with different excitation and emission filters. (**b**) Fluorescence efficiency of grafted cells, represented as the signal-to-noise ratio (signal ROI/background ROI) at excitation and emission filter combinations as above. Means ± st.dev. are shown, n=15–20. ANOVA with Tukey-Kramer posthoc test was used to calculate p-values. Asterisks indicate statistically significant differences compared to Katushka2S (* p < 0.05, ** p < 0.01, *** p < 0.001, **** p < 0.0001), brace denotes similar p-values.

To assess performance of these far-red fluorescent proteins in whole body imaging, we employed the intramuscular nude mouse model essentially as described in Ref. 4. with injection of transiently transfected cells at approximately 5 mm depth. In living mice, Katushka2S produced higher fluorescence signal and better signal-to-noise ratio than other far-red fluorescent proteins analyzed at various excitation wavelengths and in both emission channels (Fig. 1, Table 3, Supplementary Table 2), except for excitation at 640 nm where all proteins produced low signal-to-noise ratios. The highest fluorescence values for Katushka2S in the “DsRed” channel were recorded using 535-nm excitation and in the “Cy5.5” channel with 605-nm excitation. However, in the latter excitation wavelength, the difference between Katushka2S and mNeptune was not significant. These data essentially match the cell culture imaging results.

### Multiplexed *in vivo* imaging with spectral unmixing

**Figure 2:**
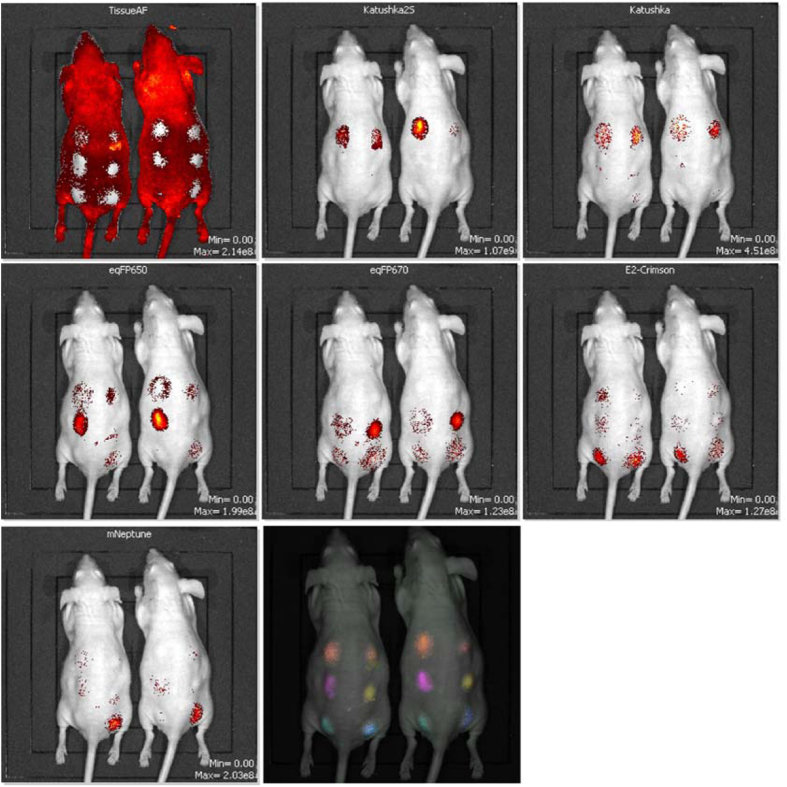
Imaging data for far-red fluorescent proteins in subcutaneous xenografts, demonstrating their feasibility for multi-label imaging. HEK293FT cells expressing transiently transfected fluorescent proteins were engrafted subcutaneously into the same mice, and imaged using IVIS Lumina II. Spectrally unmixed data and tissue autofluorescence are shown (from upper left corner: tissue autofluorescence, Katushka2S, Katushka, eqFP650, eqFP670, E2-Crimson, mNeptune, and a composite pseudocolored image).

Notably, patterns of fluorescent brightness across channels differed essentially for each fluorescent protein. For example, at shorter excitation wavelengths Katushka2S yielded significantly stronger signals than other far-red proteins. We thus asked whether the fluorescent proteins used in this study can be spectrally separated (unmixed) based on different signal-to-noise ratios in different channels, using common laboratory equipment such as an IVIS Lumina II imaging system equipped with standard filter sets. Remarkably, HEK293FT cells expressing different fluorescent proteins injected subcutaneously into the same mouse were clearly distinguished from each other, demonstrating the possibility of multiplexed *in vivo* imaging using available far-red fluorescent proteins with similar spectral characteristics (Fig. 2).

### Katushka2S versus iRFP720

**Figure 3:**
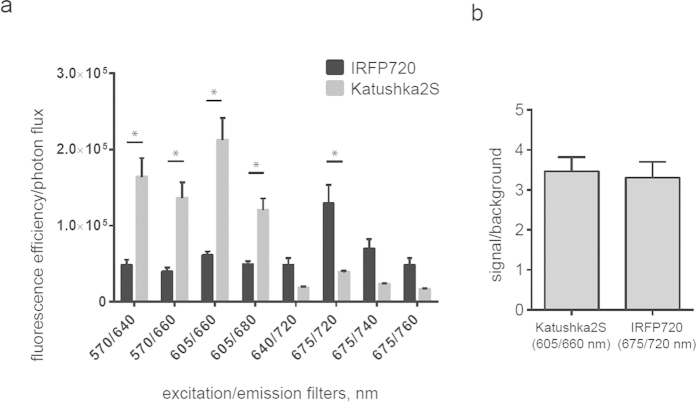
*In vivo* imaging data for Katushka2S and iRFP720 in mice. Transfected cells were injected into backs of NSG mice (500,000 and 250,000 cells each, n = 8) and imaged on IVIS Spectrum. Fluorescence signals for each implant were normalized to corresponding firefly luciferase luminescence. (**a**) Pairwise comparisons of the signals from the two different proteins at different excitation/emission wavelengths. * P < 0.0005 (t-test, statistical significance was determined using the Holm-Sidak method to correct for multiple comparisons, with alpha = 5.000%). (**b**) Signal-to-noise ratios at optimal excitation and emission wavelengths for each protein.

In spite of the notable progress in development of GFP-like far-red fluorescent proteins, potentially even better genetically encoded fluorescent labels have been recently developed on the basis of bacterial phytochrome photoreceptors^8^. To compare Katushka2S with one of the best reporters of the latter type, iRFP720, we injected HEK293T cells co-expressing firefly luciferase and either Katushka2S or iRFP720 into backs of mice at a depth of 4-5 mm. Figure 3a shows excitation/emission data across several wavelengths. Katushka2S produced brighter fluorescence intensity (all data were normalized to firefly luciferase signal) at its optimum conditions as compared with the best output from iRFP720. At the same time, both proteins showed comparable signal-to-noise ratios at the optimum wavelengths for excitation and emission (Fig. 3b) since autofluorescence from mouse tissue was lower in the channel optimal for iRFP720.

### Katushka2S versus mCardinal and mNeptune2.5

**Figure 4:**
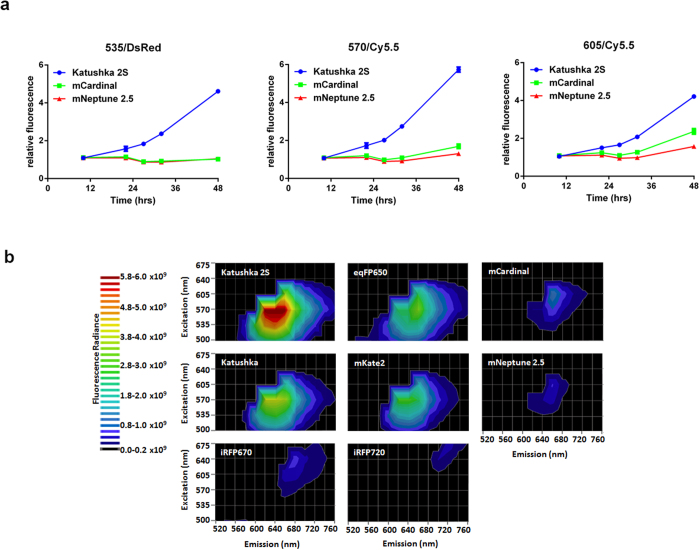
Imaging far-red and near-infrared fluorescent proteins in cells. (**a**) Fluorescence intensity of transiently transfected HEK293T cells expressing Katushka2S, mCardinal, or mNeptune2.5 was measured over time using listed excitation and emission settings on an IVIS Lumina system. Graphs show mean values ± SEM when larger than the symbol for fluorescence intensity at each time point. Fluorescence from each protein was normalized to background fluorescence from untransfected cells. (**b**) HEK293T cells transiently transfected with listed fluorescent proteins were imaged and fluorescence radiance intensity quantified on an IVIS Spectrum 48 hours after transfection. Contour plots show data for various pairs of excitation and emission filters based on the depicted pseudocolor scale.

We further performed experiments to compare Katushka2S with the recently published far-red fluorescent proteins mCardinal and mNeptune2.5^6^. In cell-based assays, Katushka2S fluorescence developed detectable signal by 12 hours following transient transfection both by macroscopic and microscopic fluorescence imaging (Fig. 4a). Fluorescence matured much slower for the other two proteins with no detectable signal until 48 hours. Macroscopic fluorescence intensity of cells expressing Katushka2S was notably higher than that of mNeptune2.5 and mCardinal at a variety of excitation-emission wavelengths combinations. Katushka2S also produced greater fluorescence intensity at the slightly longer excitation and emission wavelengths optimal for the other two proteins. Additionally, in these experiments, maximum fluorescence from Katushka2S was greater than that of the closest homologues: Katushka, mKate2, and eqFP650, and of near infrared bacterial phytochromes, iRFP670 and iRFP720 (Fig. 4b). Notably, we measured minimal and no fluorescence from Katushka2S at optimal excitation and emission wavelengths for iRFP670 and iRFP720, respectively, supporting combined use of Katushka2S and iRFP720 for dual color imaging.

**Figure 5:**
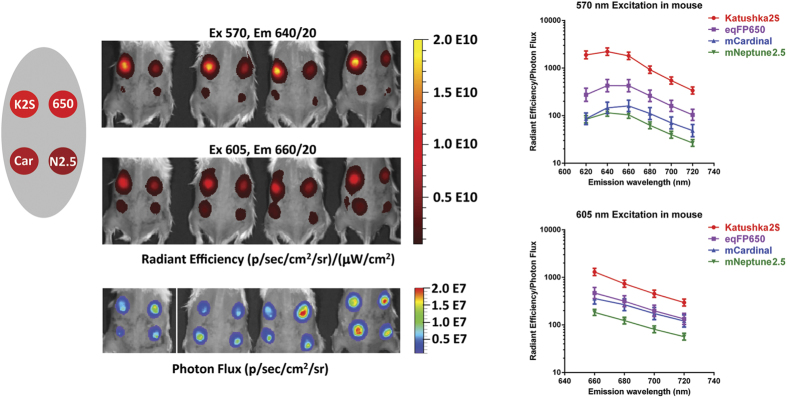
Whole animal fluorescence imaging for selected far-red fluorescent proteins. HEK293T cells were transiently co-transfected with the listed far-red fluorescent proteins (Katushka2S, K2S; eqFP650, 650; mCardinal, Car; mNeptune2.5, N2.5) and firefly luciferase. 48 hours after transfection, equal numbers of cells (2 × 10^5^ per implant) were implanted at 5 mm depth in backs of NSG mice from which hair was removed. Fluorescence was imaged on an IVIS Spectrum at excitation and emission wavelengths of 570/640 and 605/660 and quantified as radiant efficiency displayed on a pseudocolor scale. Following fluorescence imaging, bioluminescence from firefly luciferase was measured with data depicted as a pseudocolor scale for photon flux. The white vertical line separating the first and second mice from the left designates that the image of the first mouse was acquired separately from the remaining three and then presented as a group. Graphs display mean values ± SEM for fluorescence radiant efficiency normalized to luciferase photon flux for each implant (n = 4 per condition) for 570- and 605-nm excitation and listed emission filters.

To compare performance of Katushka2S, eqFP650, mNeptune2.5 and mCardinal for whole animal imaging, we injected cells co-transfected with fluorescent protein and firefly luciferase expression plasmids at approximately 5 mm depth into backs of mice. At both 570- and 605-nm excitation wavelengths, Katushka2S produced greater fluorescence at emission wavelengths up to 720 nm (Fig. 5). For 570-nm excitation, eqFP650 exhibited second highest signal, while this protein and mCardinal were comparable at 605-nm excitation. We measured lowest fluorescence from mNeptune2.5 under all conditions.

### Influence of the cryptic splice site

In direct comparison of Katushka, Katuhska2 (with cryptic splice site), and Katushka2S (without splice site), the latter two variants demonstrated equal 40% higher brightness in transiently transfected HEK293T cells (Supplementary Fig. 2). Thus higher fluorescence of cells transfected with Katushka2 or Katushka2S relative to those transfected by Katushka results from their brighter intrinsic fluorescence, while elimination of cryptic splice site did not influence fluorescent signal in cells. However, presence of cryptic splice sites in fluorescent protein-encoding sequences could play a negative role in experimental models where it results in undesirable splicing – a problem which was recognized long ago^12^. In the case of donor splice site, artifacts can arise if acceptor splice sites are situated downstream of Katushka. For example, a cryptic acceptor spice site could accidentally occur in C-terminal fusions or IRES-based constructs. Also, insertion of fully functional introns in different parts of gene is often used to enhance gene expression^13^,^14^,^15^,^16^. Finally, fluorescence reporters of alternative splicing and nonsense-mediated mRNA decay are based on transcripts which include both fluorescent protein-coding region(s) and intron(s)^17^,^18^,^19^,^20^. Thus elimination of the cryptic slice site makes Katushka2S a more robust tag for the use in different possible applications. It should be noted that mNeptune, mNeptune2.5 and mCardinal, which are highly homologous to Katushka, do not contain this cryptic splice site, whereas eqFP650 and eqFP670 do (Supplementary Fig. 3). In theory, presence of the cryptic splice site could influence comparison of the latter proteins with Katushka2S in experiments that involve bi-cistronic IRES-luciferase constructs (Figs. 1, 2 and Supplementary Fig. 1), due to the possibility of introducing a complimentary acceptor splice site. However, similar domination of Katushka2S fluorescent signal over that of eqFP650 was also observed in co-transfection experiments with single-cistron constructs (Figs. 4, 5). At the same time, eqFP670 signal yielded to that of eqFP650 in this study and our previous^4^ work.

## Discussion

Overall, our cell-based and animal data using transiently transfected cell implants establish Katushka2S as the preferable GFP-like far red fluorescent protein currently available. At the same time, the bacterial phytochrome-based fluorescent labels, such as iRFP720, reach comparable efficiency in mouse imaging. These two proteins can be combined efficiently as a far-red/near infrared pair for dual color whole body fluorescence imaging studies to track two different cell populations or molecular signals. Importantly, the two proteins are non-homologous and thus can be expressed in the same cell without concerns for homologous recombination events.

The monomeric state of mNeptune2.5, mCardinal, and mKate2 is the primary advantage of these proteins, making them suitable for localization studies in fusion with various proteins of interest in contrast to the dimeric or tetrameric Katushka2S, eqFP650, eqFP670, and E2-Crimson. In this respect, higher brightness of Katushka2S indicates that some progress is still possible in development of brighter versions of existing monomeric far-red GFP-like fluorescent proteins. However, looking to the future, the progress in development of GFP-like far-red fluorescent proteins has probably almost reached the physical limit determined by the chromophore environment^21^,^22^. Therefore, we should more likely expect further progress in development of alternative genetically encoded fluorescent labels such as bacterial phytochromes, for which evolution *in vitro* has only started.

## Methods

### Characterization of fluorescent proteins *in vitro*

Each recombinant protein with N-terminal polyhistidine tag was expressed in *E. coli* XL1 Blue strain (Invitrogen) and purified using Talon metal-affinity resin (Clontech). All measurements were performed in 20 mM Tris/HCl (pH 7.5) and 100 mM NaCl. Varian Cary Eclipse fluorescence spectrophotometer was used for measuring excitation and emission spectra. Fluorescence pH stability, fluorescence quantum yield and molar extinction coefficient were determined as described earlier^9^. Brightness was calculated as the product of the fluorescence quantum yield and molar extinction coefficient.

To compare maturation rates of each protein, transformed *E. coli* cells (XL1 Blue strain) were grown overnight in LB with 500 mg/l ampicillin and 3% D-glucose. Tubes were filled to the rim and sealed upon 1 h of induction to create an anaerobic environment. 0.4 mM IPTG was added and proteins were expressed under these anaerobic conditions for 1 h at 37 °C. Proteins were purified rapidly at 4 °C and maturation kinetics were measured by using the Varian Cary Eclipse Fluorescence Spectrophotometer with the chamber temperature maintained at 37 °C. Maturation was initiated following dilution in the renaturation buffer (35 mM KCl, 2 mM MgCl2, 50 mM Tris pH 7.5, 1 mM dithiothreitol).

Photostability was compared using an epifluorescent Leica AFLX 6000 microscope. Proteins with polyhistidine tags were bound to Talon metal affinity resin beads (Clontech), placed on a glass slide and exposed to light. For bleaching, a TexasRed filter was used with 63× oil-immersion objective, 1.5 s per image scan rate, and maximal power.

### Cell imaging

Fluorescent proteins were expressed as single-cistronic constructs in the mammalian pCMV plasmid backbone, and normalization for differences in transfection efficiencies was achieved by co-transfection of a luciferase expression plasmid (experiments described in Fig. 4). In other set of experiments (shown in Supplementary Fig. 1), E2-Crimson, mNeptune, eqFP650, eqFP670, Katushka, and Katushka2S were expressed as bi-cistronic constructs equipped with the downstream IRES-luciferase cassette, serving as an internal normalization control. Plasmids were transiently transfected into HEK293FT cells and after three days in culture, fluorescence and bioluminescence imaging was performed with *in vivo* imaging system IVIS Lumina II or IVIS Spectrum (CaliperLS, now PerkinElmer) using Living Image 4.3 software. To compare performance of fluorescent proteins in cell culture, radiant efficiency of transfected 24-well plates (with their lids removed) was determined with different excitation and emission filter sets. For bioluminescence analysis, D-luciferin (Regis Technologies, Illinois, USA) was added to the medium at concentration of 30 μg/ml and peak luminescence values were recorded. Fluorescence data (mean ± st.dev., n = 6) were reported as signal-to-noise ratio, corrected for background and normalized for transfection efficiency. This was obtained by subtracting the average fluorescent background (measured from the empty vector-containing wells) from the fluorescent flux of each fluorescent protein-containing well, divided by its luminescence and average fluorescent background ((Signal – AVE Bkg)/(Luc × AVE Bkg)).

### Whole body imaging

All animal procedures were approved by the University of Michigan Committee for Use and Care of Animals and the Animal Welfare Committee of Estonian Ministry of Agriculture. Animal care was provided in accordance with the principles and procedures outlined in the National Research Council Guide for the Care and Use of Laboratory Animals. HEK293FT cells transfected by vectors expressing a fluorescent protein and IRES-driven luciferase were also analyzed in live nude (nu/nu) mice engrafted either subcutaneously (Fig. 2), or about 5 mm deep into their gluteal musculature (Fig. 1), essentially as described^4^. Transfected cells were harvested by trypsin, counted and normalized to transfection efficiency prior to engraftment. Adjustment of all cell populations to the same transfection level was done according to their luciferase activity, measured from diluted samples with IVIS as described above. For intramuscular engraftment, approximately 5 × 10^6^ cells in 100 μl F12 medium/Matrigel mixture (1:1) were injected per site into isoflurane-sedated mice, and 1–2 hrs post-injection fluorescent images were acquired with IVIS. For studies with NSG mice, we used 2 × 10^5^ cells. Fluorescence data (mean ± st.dev., n = 15–20) were reported as signal-to-noise ratio (Signal/Bkg), where background fluorescence was measured from the same mouse nearby the injection site. In subcutaneous implantation experiments, each mouse was implanted simultaneously with six different fluorescent protein-expressing cells (n = 4–5). 2.5 ×10^6^ cells in 50 μl F12 medium/Matrigel mixture (1:1) were injected per site.

Experiments comparing Katushka2S with iRFP720 or mCardinal, mNeptune2.5, and eqFP650, shown on Figs. 3, 5 were performed similarly. Co-transfection of a luciferase expression plasmid was used instead of bi-cistronic IRES constructs. 2 × 10^5^ cells were injected 4–5 mm deep into back muscles of NSG mice (Jackson Laboratory) (n = 4–8). After fluorescence measurements, bioluminescence analysis was performed. Awake animals were injected i.p. with D-luciferin at 150 mg/kg body weight, isoflurane-sedated and the peak luminescence values per each injection were recorded. Background fluorescence for each filter combination was determined from two uninjected mice. Statistical analysis was performed using SAS JMP10.0. To compare means, oneway ANOVA with Tukey-Kramer post hoc test was used.

## Additional Information

**How to cite this article**: Luker, K.E. *et al*. Comparative study reveals better far-red fluorescent protein for whole body imaging. *Sci. Rep.* **5**, 10332; doi: 10.1038/srep10332 (2015).

## Supplementary Material

Supplementary Information

Supplementary Information

Supplementary Information

## Figures and Tables

**Table 1:** Characteristics of GFP-like far-red fluorescent proteins.

	**eqFP670**	**E2-Crimson**	**mNeptune**	**eqFP650**	**mCardinal**	**Katushka**	**mNeptune2.5**	**Katushka2S**
Excitation peak (nm)	605	605	599	592	604^d^	588	599^d^	588^b^
Emission peak (nm)	670	646	649	650	659^d^	635	643^**d**^	633^b^
Fluorescence quantum yield	0.06	0.12	0.18	0.24	0.19^d^	0.34	0.28^d^	0.44^b^
Molar extinction coefficient (М^−1·^cm^−1^) at excitation maximum	70,000	58,500	57,500	65,000	87,000^d^	65,000^b^	95,000^d^	67,000^b^
Brightness (a.u.)^a^	4,200	7,080	10,350	15,600	16,530^d^	22,100	26,600^d^	29,480^b^
Maturation half-time (min)^c^	ND	ND	18^b^	18^b^	27^d^	25^b^	26^d^	14^b^
Photostability, widefield (s)	1420^b^	46^b^	500^b^	404^b^	ND	208^b^	ND	72^b^
pKa	4.5	4.5	5.8	5.7	5.8^d^	5.5	5.3^d^	5.4^b^
References	4	3	2	4	6	1	6	this work

^a^product of fluorescence quantum yield and molar extinction coefficient.

^b^measured in this work. Katushka2S fluorescence quantum yield was determined relative to Katushka.

^c^eqFP670 and E2-Crimson do not mature *in vitro* in our assay.

^d^data from Ref. 6.

**Table 2:**
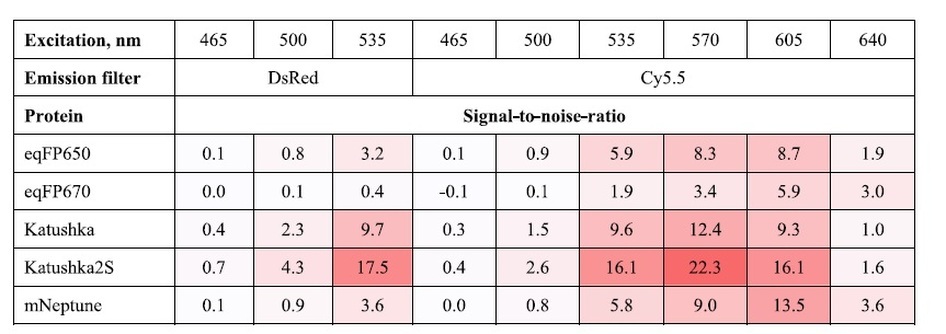
Comparison of signal-to-noise-ratios of far-red FPs imaged in cell culture with IVIS Lumina II standard filter sets.

**Table 3:**
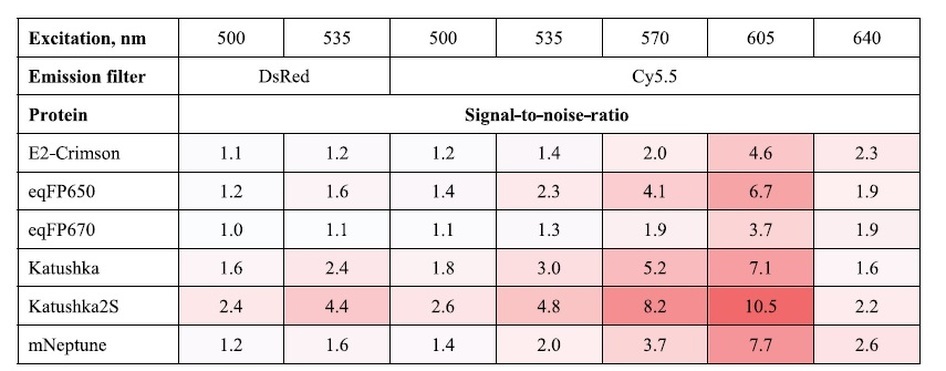
Comparison of signal-to-noise-ratios of far-red FPs imaged in intramuscular mouse model with IVIS Lumina II standard filter sets.

## References

[b1] Shcherbo D. *et al.* Bright far-red fluorescent protein for whole-body imaging. Nat Methods 4, 741–746 (2007). 17721542 10.1038/nmeth1083

[b2] Lin M. Z. *et al.* Autofluorescent proteins with excitation in the optical window for intravital imaging in mammals. Chem Biol. 16, 1169–1179 (2009). 19942140 10.1016/j.chembiol.2009.10.009 PMC2814181

[b3] Strack R. L. *et al.* A rapidly maturing far-red derivative of DsRed-Express2 for whole-cell labeling. Biochemistry 48, 8279–8281 (2009). 19658435 10.1021/bi900870u PMC2861903

[b4] Shcherbo D. *et al.* Near-infrared fluorescent proteins. Nat Methods 7, 827–829 (2010). 20818379 10.1038/nmeth.1501 PMC4425247

[b5] Luker K. E. *et al.* *In vivo* imaging of ligand receptor binding with Gaussia luciferase complementation. Nat Med. 18, 172–177 (2012). 22138753 10.1038/nm.2590 PMC3253890

[b6] Chu J. *et al.* Non-invasive intravital imaging of cellular differentiation with a bright red-excitable fluorescent protein. Nat Methods 11, 572–578 (2014). 24633408 10.1038/nmeth.2888 PMC4008650

[b7] Shu X. *et al.* Mammalian expression of infrared fluorescent proteins engineered from a bacterial phytochrome. Science 324, 804–807 (2009). 19423828 10.1126/science.1168683 PMC2763207

[b8] Shcherbakova D. M. & Verkhusha V. V. Near-infrared fluorescent proteins for multicolor *in vivo* imaging. Nat Methods 10, 751–754 (2013). 23770755 10.1038/nmeth.2521 PMC3737237

[b9] Shcherbo D. *et al.* Far-red fluorescent tags for protein imaging in living tissues. Biochem J. 418, 567–574 (2009). 19143658 10.1042/BJ20081949 PMC2893397

[b10] Gurskaia N. G., Staroverov D. B., Fradkov A. F. & Luk’ianov K. A. [Coding region of far-red fluorescent protein katushka contains a strong donor splice site]. Bioorg Khim. 37, 425–428 (2011). 21899059 10.1134/s1068162011030071

[b11] Leblond F., Davis S. C., Valdes P. A. & Pogue B. W. Pre-clinical whole-body fluorescence imaging: Review of instruments, methods and applications. J Photochem Photobiol B. 98, 77–94 (2010). 20031443 10.1016/j.jphotobiol.2009.11.007 PMC3678966

[b12] Haseloff J., Siemering K. R., Prasher D. C. & Hodge S. Removal of a cryptic intron and subcellular localization of green fluorescent protein are required to mark transgenic Arabidopsis plants brightly. Proc Natl Acad Sci USA 94, 2122–2127 (1997). 9122158 10.1073/pnas.94.6.2122 PMC20051

[b13] Buchman A. R. & Berg P. Comparison of intron-dependent and intron-independent gene expression. Mol Cell Biol. 8, 4395–4405 (1988). 3185553 10.1128/mcb.8.10.4395-4405.1988 PMC365513

[b14] Lacy-Hulbert A. *et al.* Interruption of coding sequences by heterologous introns can enhance the functional expression of recombinant genes. Gene therapy. 8, 649–653 (2001). 11320412 10.1038/sj.gt.3301440

[b15] Moabbi A. M., Agarwal N., El Kaderi B. & Ansari A. Role for gene looping in intron-mediated enhancement of transcription. Proc Natl Acad Sci USA 109, 8505–8510 (2012). 22586116 10.1073/pnas.1112400109 PMC3365183

[b16] Pereverzev A. P. *et al.* Intron 2 of human beta-globin in 3′-untranslated region enhances expression of chimeric genes. Rus. J. Bioorgan. Chem. 40, 269–271 (2014). 10.1134/s106816201403011x 25898735

[b17] Wang Z. *et al.* Systematic identification and analysis of exonic splicing silencers. Cell 119, 831–845 (2004). 15607979 10.1016/j.cell.2004.11.010

[b18] Paillusson A., Hirschi N., Vallan C., Azzalin C. M. & Muhlemann O. A GFP-based reporter system to monitor nonsense-mediated mRNA decay. Nucleic Acids Res. 33, e54 (2005). 15800205 10.1093/nar/gni052 PMC1072805

[b19] Kuroyanagi H., Kobayashi T., Mitani S. & Hagiwara M. Transgenic alternative-splicing reporters reveal tissue-specific expression profiles and regulation mechanisms *in vivo*. Nat Methods 3, 909–915 (2006). 17060915 10.1038/nmeth944

[b20] Gurskaya N. G. *et al.* Analysis of alternative splicing of cassette exons at single-cell level using two fluorescent proteins. Nucleic Acids Res. 40, e57 (2012). 22259036 10.1093/nar/gkr1314 PMC3333876

[b21] Pletnev S. *et al.* Structural basis for bathochromic shift of fluorescence in far-red fluorescent proteins eqFP650 and eqFP670. Acta Crystallogr D Biol Crystallogr. 68, 1088–1097 (2012). 22948909 10.1107/S0907444912020598 PMC3489099

[b22] Pletneva N. V. *et al.* Crystallographic study of red fluorescent protein eqFP578 and its far-red variant Katushka reveals opposite pH-induced isomerization of chromophore. Protein Sci. 20, 1265–1274 (2011). 21563226 10.1002/pro.654 PMC3149199

