# Supplementary material for: Comparative study reveals better far-red fluorescent protein for whole body imaging
Source: Sci Rep. 2015 Jun 2;5:10332. doi: 10.1038/srep10332 (PMC4603699; doi:10.1038/srep10332)
Supplement: Supplementary Information [file srep10332-s1.pdf]

Supplementary

Comparative study reveals better far-red fluorescent protein for whole body imaging

Luker K.E.[1]*, Pata P.[2*], Shemiakina I.I.[3,4*], Pereverzeva A.[3*], Stacer A.C.[1], Shcherbo D.S.[3,4], Pletnev V.Z.[3], Skolnaja M.[2], Lukyanov K.A.[3,5], Luker G.D.[1], Pata I.[2], Chudakov D.M.[3,6,7**]

[1]Department of Radiology, University of Michigan Medical School, Ann Arbor, MI 48109-2200, USA;
[2]Tallinn University of Technology, Department of Gene Technology. 15 Akadeemia St, Tallinn 12618, Estonia.
[3]Shemiakin-Ovchinnikov Institute of Bioorganic Chemistry, Russian Academy of Science, Miklukho-Maklaya 16/10, 117997, Moscow, Russia;
[4]Evrogen JSC, Miklukho-Maklaya 16/10, 117997, Moscow, Russia.
[5]Nizhny Novgorod State Medical Academy, Nizhny Novgorod, Russia.
[6]CEITEC MU, Masaryk University, Brno, Czech republic.
[7]Pirogov Russian National Research Medical University, 117997 Moscow, Russia

* contributed equally

**corresponding author

Supplementary Tables

Supplementary Table 1. Comparison of signal-to-noise ratios of far-red fluorescent proteins imaged in cell culture with IVIS Lumina II standard filter sets.

Supplementary Table 2. Comparison of signal-to-noise ratios of far-red fluorescent proteins imaged in intramuscular mouse model with IVIS Lumina II standard filter sets.

Supplementary Figures

Supplementary Figure 1. Cell-based imaging of transiently transfected far-red fluorescent proteins in HEK293FT cells, using an IVIS Lumina II.

Supplementary Figure 2. Flow cytometry analysis of HEK293T cells transiently transfected with Katushka, Katushka2, and Katushka2S.

Supplementary Figure 3. Nucleotide alignment of Katushka and closely homologous fluorescent protein genes at the location of cryptic donor splice site.

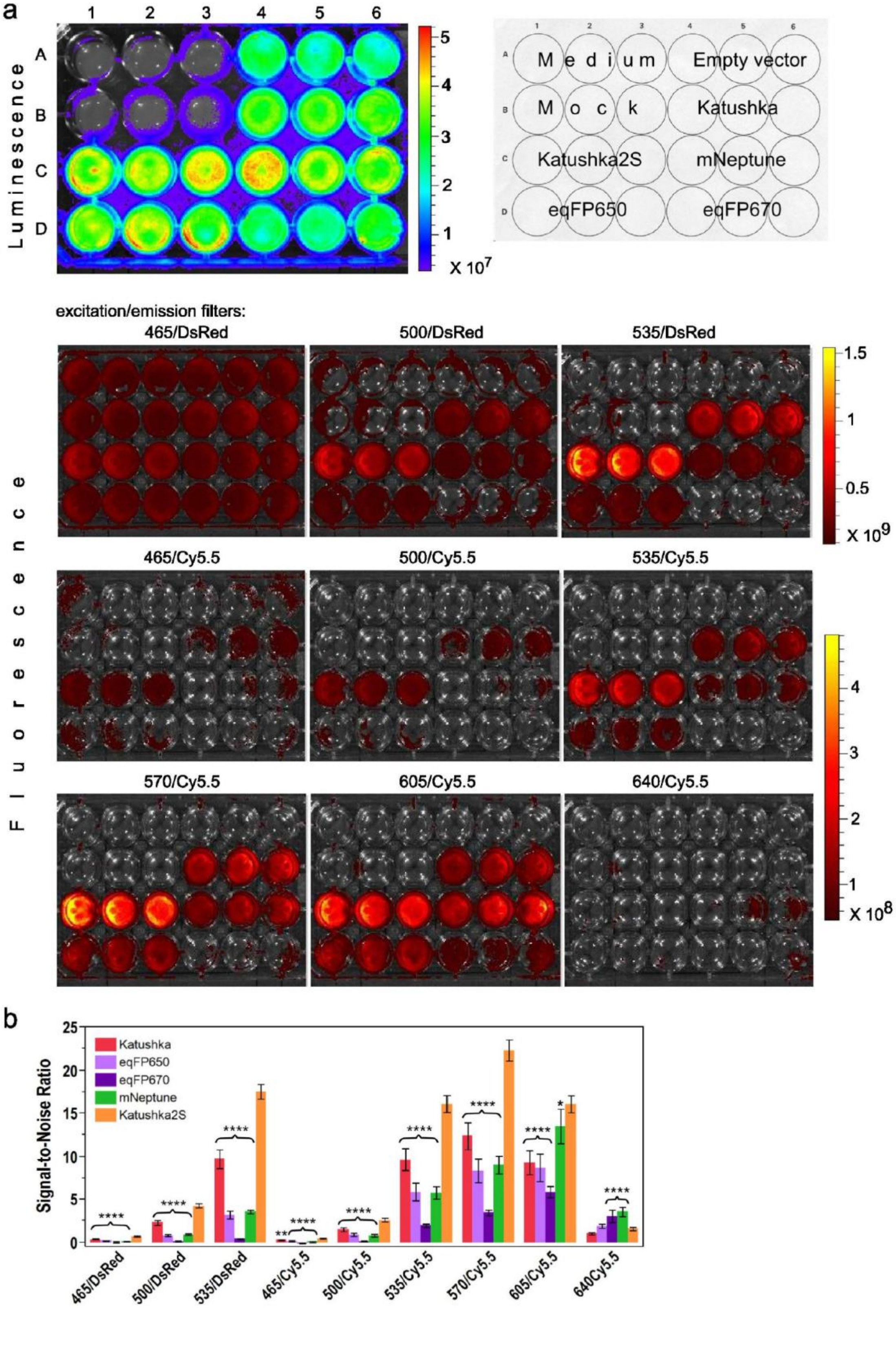
a
Luminescence
Medium
Empty vector
Mock
Katushka
Katushka2S
mNeptune
eqFP650
eqFP670
X 10^7
excitation/emission filters:
465/DsRed
500/DsRed
535/DsRed
X 10^9
Fluorescence
465/Cy5.5
500/Cy5.5
535/Cy5.5
570/Cy5.5
605/Cy5.5
640/Cy5.5
X 10^8
b
Signal-to-Noise Ratio
Katushka
eqFP650
eqFP670
mNeptune
Katushka2S
465/DsRed
500/DsRed
535/DsRed
465/Cy5.5
500/Cy5.5
535/Cy5.5
570/Cy5.5
605/Cy5.5
640Cy5.5

Supplementary Figure 1. **Cell-based imaging of transiently transfected far-red fluorescent proteins in HEK293FT cells.** (**a**) Representative luminescence and fluorescence images of multiwell plates with HEK293FT cells expressing Katushka, eqFP650, eqFP670, mNeptune or Katushka2S, captured with indicated excitation and emission filter combinations **on IVIS Lumina II**. Excitation filters are of 35-nm bandwidth centered at the wavelength indicated, and emission filters DsRed and Cy5.5 are 575-650 and 695-770 nm, respectively. (**b**) Data presented as fluorescence signal-to-noise ratios (normalized signal ROI/background ROI) at given excitation wavelength (nm) and emission filter combinations. Signal ROI was normalized for transfection efficiency by the IRES-driven luciferase activity included in the vector, and background was defined as the average fluorescence of cells transfected with an empty vector. Means ± st.dev. are shown, n=6. ANOVA with Tukey-Kramer posthoc test was used to calculate p-values. Asterisks indicate statistically significant differences compared to Katushka2S (* p<0.05, ** p<0.01, *** p<0.001, **** p<0.0001), brace denotes similar p-values.

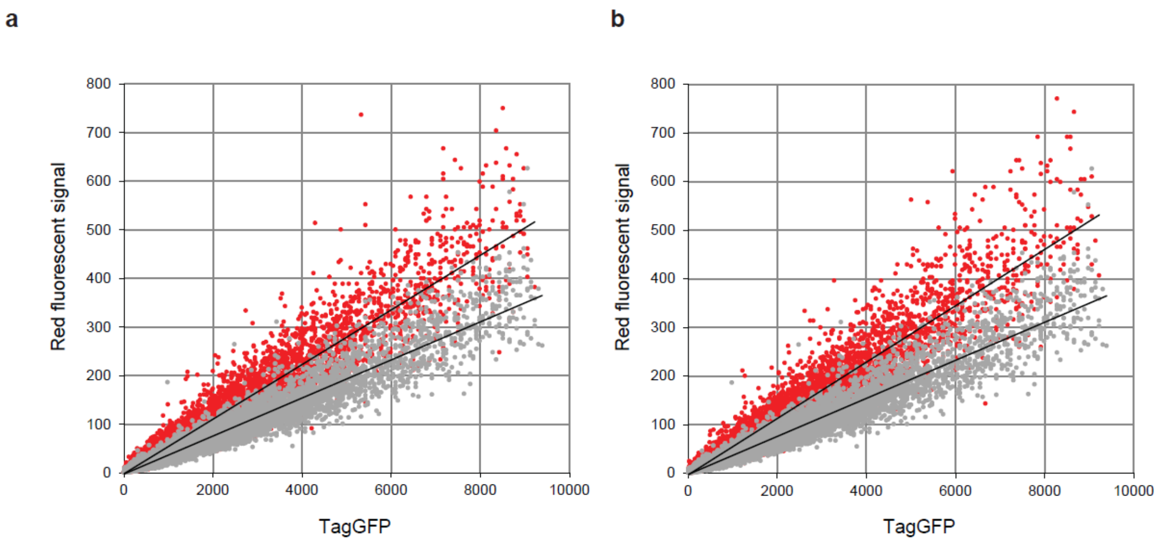

c

**Transfection experiment**	**Red fluorescent signal**	**Normalized red fluorescent signal**
Katushka, replica 1	6.5	6.0
Katushka, replica 2	6.7	6.0
Katushka2, replica 1	8.2	8.3
Katushka2, replica 2	8.8	8.4
Katushka2S, replica 1	8.0	8.4
Katushka2S, replica 2	8.7	8.6

Supplementary Figure 2. Flow cytometry analysis of HEK293T cells transiently transfected with Katushka, Katushka2 (with splice site), and Katushka2S (without splice site). Cells were cotransfected with TagGFP-N vector (Evrogen) for normalization. **a**. Katushka2 (red) and Katushka (gray) signals are shown as overlay. **b**. Katushka2S (red) and Katushka (gray) signals are shown as overlay. **c**. Fluorescence efficiency and normalized fluorescence efficiency in replicate transfection experiments.

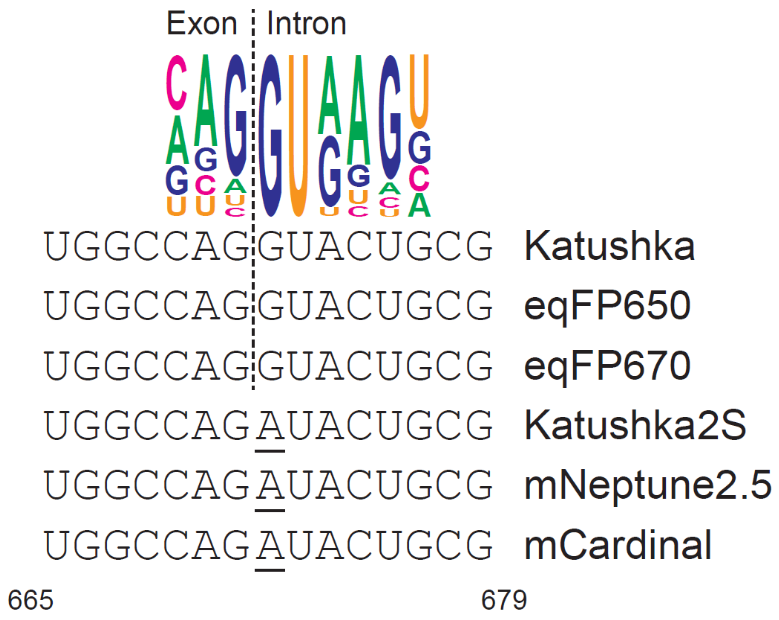

Supplementary Figure 3. Nucleotide alignment of Katushka and closely homologous fluorescent protein genes at the location of cryptic donor splice site. General donor splice site consensus (adapted from Ref. 1) is shown on the top. Mutation eliminating splice site is underlined. Nucleotide positions counting from the first ATG are shown with numbers.

Reference

1. Cartegni, L., Chew, S.L. & Krainer, A.R. Listening to silence and understanding nonsense: exonic mutations that affect splicing. *Nature reviews. Genetics* **3**, 285-298 (2002).
